# Pain could negatively affect school grades - Swedish middle school students with low school grades most affected

**DOI:** 10.1371/journal.pone.0208435

**Published:** 2018-12-06

**Authors:** Anna Grimby-Ekman, Maria Åberg, Kjell Torén, Jonas Brisman, Mats Hagberg, Jeong-Lim Kim

**Affiliations:** 1 Health Metrics, Public Health and Community Medicine, Sahlgrenska Academy, University of Gothenburg, Gothenburg, Sweden; 2 Occupational and environmental medicine, Public Health and Community Medicine, Sahlgrenska Academy, University of Gothenburg, Gothenburg, Sweden; 3 Department of Public Health and Community Medicine/Primary Health Care, Institute of Medicine, at Sahlgrenska Academy, University of Gothenburg, Gothenburg, Sweden; Indiana University, UNITED STATES

## Abstract

Recurrent headache, abdominal and musculoskeletal pain are common in adolescents and it is therefore important to understand their impact on the transitional period from childhood to adulthood. However, studies of the prevalence over time and implications on educational outcomes are still limited, especially regarding multiple pain symptoms. The present study material consists of questionnaire surveys, completed in 2000 and 2008, including two study populations of 9th grade adolescents aged 15 living in West Sweden (n = 20 877). Pain symptoms and demographic variables were based on self-reports from the questionnaires, and school grades were obtained from Statistics Sweden after the student had finished their 9th grade. Between 2000 and 2008, the prevalence of abdominal pain increased among Swedish adolescents (largest increase in girls); the prevalence of headache increased only in girls; the prevalence of pain in upper body decreased only in boys. School grades were significantly lower among those with headache or abdominal pain. Among students with low school grades (10th percentile) the estimated difference between those having any of the symptoms or none were -27 school grade units (95% confidence interval for girls (-27.8; -26.0), for boys (-27.6; -25.5). Both symptoms being present pronounced the association. Low parental education increased the negative effect of symptoms on school grades, most pronounced in the group with the lowest grades. In conclusion, identification of pain symptoms may improve academic achievements, especially in students with multiple symptoms and with parents having low education. Further intervention studies are need.

## Introduction

Recurrent headache, abdominal pain and musculoskeletal pain are common symptoms in children and adolescents [1–8]. These pain symptoms are often comorbid with each other [9]. Headache has been considered as one of the most commonly occurring pain symptoms among schoolchildren [3, 8], and therefore in focus in the literature. In a Dutch population study it was reported that about 25% of children aged 4–18 had recurrent or persistent headache for more than 3 months [10]. The prevalence of headache seems to increase with age and to be higher in girls than in boys [3, 10–13]. A review study concluded that the prevalence rates of pain varied greatly not only regarding the different locations of pain, but also between cohorts and settings; the prevalence of headache ranged between 8–83% and musculoskeletal pain of 4–40% [1]. Similar finding was observed by the WHO collaborative cross-national survey among school-aged children from 28 countries [3].

In the literature, the prevalence of pain symptoms has been mostly studied to describe age related differences [1, 3] and rarely to describe differences over time in society, in groups of same age, as in the present study.

Research about pediatric pain other than headache is still limited [1, 14] and especially studies on multiple pain symptoms are even less investigated [3, 10]. One of the few studies on multiple pain symptoms showed that 50% of schoolchildren aged 9, 12 and 15 years had experienced pain, either as headache, abdominal pain or musculoskeletal pain, within the recall period, and twice as many girls than boys suffered from headache [6]. The WHO collaborative survey reported that approximately 75% had at least one of the pain symptom headache, stomachache and backache, and 35% had all three [3].

Few studies attempted to investigate the consequences of pain symptoms among schoolchildren. Especially for adolescents, the academic achievements are closely related to access to higher educational choices. Hence, it is of importance to understand possible risk factors for lower academic achievements in adolescents. There are some studies in adults, based on brain imaging, suggestion that chronic pain is related to decreased gray matter densities, and hence a possible mechanism for decreased cognitive function [15]. Even for headaches, studies directed at the disabling effects are few [16]. However, it has been reported associations between restriction in daily living, increased school absenteeism and decreased quality of life and headaches [17–19] In studies trying to capture consequences on academic achievement the measures commonly used are self-reported absenteeism, and use of objective measures on academic achievements are still limited use of. In one of the few available studies on objectively measured academic achievements it was found that headache is associated with lower quality of life and poor academic performance among children aged 10–15 years [18]. In another study, it was shown that headache was associated with lower reading scores even after adjusting for the presence of emotional symptoms [20]. A study with relatively large study population aged 12 years showed that socio-economic status and recent life events were associated with poor academic performance but not directly by recurrent abdominal pain [14].

Factors considered associated with pain symptoms, among adolescence, are sedentary behavior, sleep habits and stress level. In a population-based study of adolescents, it was shown that a moderate physical activity level was associated with reduced odds for neck and shoulder pain, as well as for low back pain, compared with a low level of physical activity (exercise for only one day/week or less) [21]. Demographic factors, such as parental education and socioeconomics, are considered associated with pain in children and adolescents. A few studies have showed that frequent headache was more common in children from families with low socioeconomic status [22, 23]. The risk for chronification of pain in children is highest among girls and in children with foreign origin, emotional distress and frequent pain episodes [8].

Since studies on the prevalence of multiple pain symptoms among schoolchildren and the possible implications on educational outcome are still limited, the aims of the present study were to quantify the prevalence of different pain symptoms among adolescents over time, the co-occurrence of symptoms, and associations of these symptoms with school grades.

Specific research questions:

- How prevalent are headache, abdominal and upper body pain among schoolchildren in ninth grade, and have the prevalence increased between the years 2000 and 2008?
- Is the presence of these symptoms, or their combination, associated with final school grades?
- Is the potential association, between symptoms and school grades, modified by parental education?

## Materials and methods

The current study was undertaken using two previous large cross-sectional studies: “Allergy 2000 (All000)” from year 2000 and “Youth in Västra Götaland County” from year 2008. Detailed descriptions of the methods have been published elsewhere [24–27]. In brief, both of the previous studies were carried out in all 49 municipalities in Västra Götaland, the third largest county in Sweden, with 1.5 million inhabitants. A self-administered questionnaire was mailed to all schoolchildren at ninth grade, who were living in the county in the autumn of 2000 and repeated in same fashion in 2008. The questionnaire includes mainly respiratory related items, but also questions on pain symptoms and health in general. The questionnaires were returned by 10837 subjects in year 2000 and by 11754 in year 2008 (response rate 59.7% and 54.3% respectively).

Information on the final academic achievements after completion of their ninth grade, parents’ education and descent of each subject who had completed the questionnaire and given their full personal identification number was obtained from Statistics Sweden (Statistiska Centralbyrån, SCB). Subjects who had not given their full personal identification number were excluded from the current analysis (1031 subjects in 2000 and 1035 subjects in 2008). Only those who answered to all three questions on outcomes, i.e. pain in upper body, headache and abdominal pain, were included in the study population. Consequently, the study population consisted of 9503 schoolchildren in 2000 and 11374 in 2008. The study was approved by the Ethics Committee of the University of Gothenburg approval no. 279–00 and 471–07) and the Secrecy Clearance at SCB, allowing us to obtain consent from parents or guardians of the minors directly through the participation of the questionnaire survey.

### Definition of the pain variables

The pain variables used in this study were based on questions with only two possible answer categories, yes and no.

*Pain in upper body* was defined as 1 = yes, ever had pain/ache in neck, back, arms or hands, and 0 = no. *Headache* was defined as 1 = yes, have often headache, and 0 = no. *Abdominal pain* was defined as 1 = yes, have often pain in the stomach, and 0 = no.

These pain variables were used as outcome variables in the estimation of prevalence over the years. *Headache* and *abdominal pain* was also used in the analyses of association between symptoms and school grades together with *Pain symptoms* and *Pain categories*, defined below. *Pain in upper body* was not used in these analyses as it assesses the lifetime prevalence. Hence, due to the phrasing of the question it was not considered as a plausible cause for the final school grades after finishing ninth grade.

*Pain symptoms* was defined as 1 = at least one of the two symptoms present, headache or abdominal pain, and 0 = neither of the two symptoms present.

*Pain categories* was defined to have four categories as follows; 1 = *no pain* (neither headache nor abdominal pain), *2 = only headache*, *3 = only abdominal pain*, and *4 = headache and abdominal pain*.

### Definition of School grades

At the time of the study the total school grade was officially calculated as the sum of 16 subjects; each subject was graded with 0, 10, 15, or 20 points. Thus, the range of possible values for the total grade was 0 to 320. Summation of grades was, at the time, the standard way in Sweden to express the cumulated knowledge of 9 years in elementary school and were used as the entrance criterion to senior high school.

### Definition of demographic variables

*Parental origin* was defined as 1 = either the child was foreign born or both parents were foreign born, and 0 = otherwise.

*Parental education* was defined as 1 = highest known education among parents were pre-high school, 2 = highest known education among parents were high school and 3 = highest known education among parents were college or university.

As different answer alternatives were used in the two questionnaires from 2000 respectively 2008 for the question about sedentary behavior, they were defined by allowing a minimal difference.

*Sedentary behavior* was, in the data from year 2000, defined as 1 = leisure physical activity (enough active to get breathless and sweaty) not more than once or twice a month or less, and in the data from year 2008 as 1 = light leisure physical activities (walking or biking) sometimes, seldom or mostly sedentary activities.

*Sedentary behavior* was, in the data from year 2000, defined as 0 = leisure physical activity, enough active to get breathless and sweaty, at least once a week, and in the data from year 2008 as light leisure physical activity (also count walking and biking to and from school) regularly at least once a week.

*Problems falling asleep* was defined as 1 = yes, have often problems falling asleep in the evening 0 = no.

### Statistical methods

The prevalence (%) of symptoms were computed together with 95% confidence intervals (CI) based on the Wilson method [28]. Estimating adjusted prevalence for each year, as well as the prevalence difference (PD) between the years, were done based on logistic regression. Adjusting prevalence of symptoms were performed using inverse probability treatment weights (IPTW) [29]. Both crude and adjusted analyses are presented. The later analyses were checked for confounding by parental education and parental origin. These estimations were all stratified for sex. Data from both 2000 and 2008 is used for estimating prevalence of symptoms over time.

The association between symptoms and school grades were analyzed using quantile regression, with logistic link function [30], to handle the that the outcome school grade is a bounded variable. This method used the total data set and should not be confused with regression on sub-samples based on quantiles of data. Quantile regression represents an alternative to traditional statistical methods, both for solving the problem with non-normally distributed data, and for answering research questions about distributional subgroups of the outcome. By estimating a regression model for a spectrum of percentiles instead of for only the mean, quantile regression generally permits greater insight on the outcome over the entire distribution of individuals in a population. In this study it enables us to look at the impact of pain symptoms, on school grades, in the specific subgroups of students: students in general having low school grades (10^th^ percentile), students in general having median school grades (50^th^ percentile) and students in general having high school grades (90^th^ percentile). This gives us the possibility to investigate specific research questions, e.g. if the group of student with low grades (10^th^ percentile) is a vulnerable group.

Potential confounders were sex, parental education and parental origin. Sedentary behavior and sleep problems could either be potential confounders or on the causal pathway between pain and grades. Therefore, adjusting for these variables, the remaining association could be interpreted either as the “direct” effect (if the determinant is on the causal pathway) or as a less confounded association (if the determinant is a confounder) between pain symptoms and school grades. When looking at the association between symptoms and school grades, data from both 2000 and 2008 were used for the analyses and the analyses were adjusted for year.

Estimates of school grades are presented together with 95% confidence intervals (CIs). These are calculated using normal approximation, and the 95% CIs for group differences of school grades are calculated using the Mover method [31].

Variables analyzed for possible effect modification were parental education, sedentary behavior and problems falling asleep. *Parental education* was dichotomized, due to few observations in some variable combinations and due to simplifying the interpretation of the results. Hence, in the interaction analysis *Parental education* was defined as

0 = highest known education among parents were either pre-high school or high school

1 = highest known education among parents were college or university.

All statistical analysis are presented as point estimates and 95% confidence intervals (CI), rather than p-values. The 95% CI can be interpreted as a test saying if the null hypothesis can be rejected at the 0.05 level or not. We chose to emphasize CIs in this way, over statistical hypothesis testing, as describing possible differences and the precision of these differences is here of more interest than p-values. The statistical software used was SAS (SAS Institute Inc., Cary, NC, USA, Version 9.3) and STATA (Version 13).

## Results

Background information of the current study participants is shown in Table 1, together with school grades.

**Table 1:** Background variables in 2000 and 2008 presented as percentages.

	Study population 2000, N = 9503	Study population 2008, N = 11 374
	%	%
**Girls**	50	51
**Boys**	50	49
**Parent education**		
Middle school	8	3
High school	47	45
College, University	45	52
**Foreign origin**	12	9
**Sedentary behavior**	19	20
**Problems falling asleep**	21	28
	Mean	Mean
	Median	Median
	Min , max	Min , max
**Final school grades**	213	220
	215	225
	0 , 320	0 , 320

Mean school grades for the whole Swedish population of students finishing ninth grade (middle school) were in 2000 for boys 192 and for girls 215 [32], while in 2008 for boys 199 and for girls 221 [33]. In the current study population, the mean level year 2000 was 203 for boys and 224 for girls; corresponding values in 2008 were for boys 210 and for girls 229.

### Prevalence of symptoms 2000 and 2008

*Pain in upper body* was the most prevalent symptom for both boys and girls, though it has to be taken into account that this symptom was assessed as experiencing pain ever, Table 2.

**Table 2:** Prevalence and difference in prevalence of symptoms among ninth grades in Western Sweden, year 2000 and 2008.

	Girls				Boys			
	Crude prevalence, %		PD, 2008–2000, %		Crude prevalence, %		PD, 2008–2000, %	
	(95% CI)		(95% CI)		(95% CI)		(95% CI)	
	2000	2008	Crude PD	Adjusted PD	2000	2008	Crude PD	Adjusted PD
	N = 4792	N = 5758	N = 10550	N = 10441	N = 4711	N = 5616	N = 10327	N = 10232
	62	64	1	1	55	50	-5	-5
**Pain in upper body** (ever)	(60.8 ; 63.6)	(62.3 ; 64.8)	(-0.5 ; 3.2)	(-0.2 ; 2.9)	(53.4 ; 56.2)	(48.2 ; 50.8)	(-7.2 ; -3.4)	(-7.0 ; -3.7)
	32	35	3	3	16	15	-1	-1
**Headache** (often)	(30.9 ; 33.5)	(33.8 ; 36.3)	(1.0 ; 4.6)	(1.6 ; 4.7)	(15.1 ; 17.2)	(14.2 ; 16.1)	(-2.4 ; 0.4)	(-2.0 ; 0.4)
	18	23	5	6	8	10	2	2
**Abdominal pain** (often)	(16.9 ; 19.1)	(22.3 ; 24.5)	(3.8 ; 6.9)	(4.4 ; 7.1)	(7.5 ; 9.1)	(9.6 ; 11.2)	(1.0 ; 3.2)	(1.2 ; 3.1)
	73	75	3	3	61	57	-3	-3
**Any of the three pain symptom present**	(71.7 ; 74.2)	(74.3 ; 76.6)	(0.9 ; 4.2)	(1.2 ; 4.0)	(59.3 ; 62.1)	(56.0 ; 58.6)	(-5.3 ; -1.5)	(-5.0 ; -1.8)
	9	11	2	2	3	3	-1	-1
**All three pain symptoms present**	(7.8 ; .4)	(10.0 ; 11.6)	(-6.3 ; 10.7)	(1.5 ; 3.4)	(2.7 ; 3.7)	(2.1 ; 3.0)	(-3.9 ; 2.5)	(-1.2 ; -0.1)

The adjusted values are according to confounder distribution year 2008. PD = prevalence difference. CI = confidence interval. Adjusted PD is adjusted for parental education and foreign origin.

The second most common pain symptom was *headache*, about twice as common among girls, Table 2. For girls the prevalence of *headache*, *abdominal pain*, having any of the three symptoms and having all of the three pain symptoms increased between the years 2000 and 2008. For boys all symptoms were less prevalent than for girls, and *pain in upper body* seemed to decrease, while *abdominal pain* seemed to increase.

The prevalence of having *any of the three pain symptoms* increased for girls and decreased for boys between year 2000 and 2008. The prevalence of having all three symptoms present was about three times more common among girls than among boys (Table 2).

Additional analyses of the change in prevalence over the eight-year period, adjusting also for sedentary behavior and problems falling asleep, did not affect the results (Adjusted PD regarding all three symptoms present: 2 with 95% CI (1.1 ; 3.1) for girls and -1 with 95% CI (-1.3 ; -0.2) for boys).

### Association of symptoms with school grades

**Fig 1:**
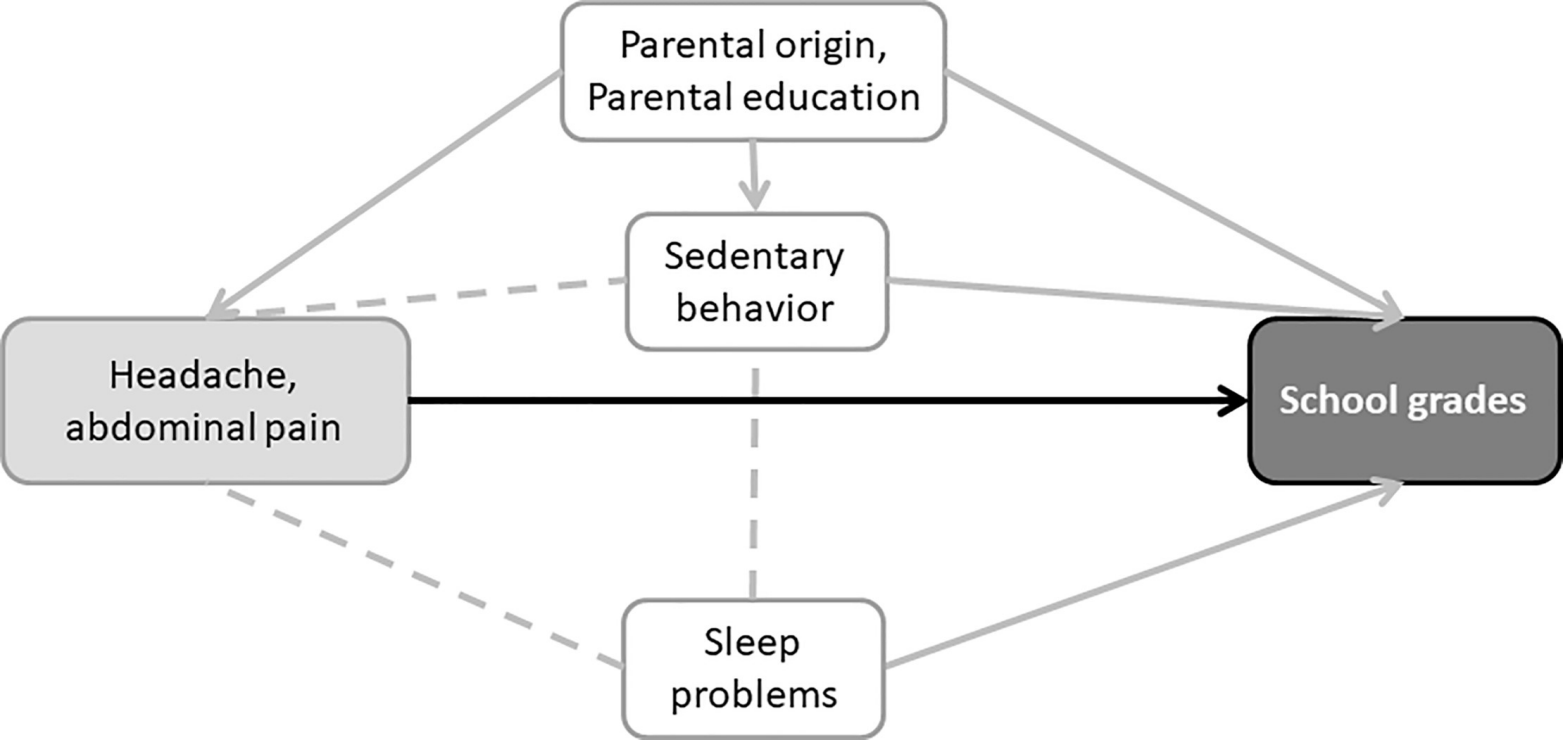
One possible theoretical model of the association between the outcome school grades and the explanatory variables pain symptoms (headache, abdominal pain). Parental origin and parental education are here confounders. The dotted lines represent undefined direction of an association, which means that sedentary behavior and sleep problems could be either confounders or mediators.

Our second aim was to investigate the impact pain symptoms might have on school grades. The outcome variables analyzed were *pain symptoms* and *pain categories*, which included combinations of the symptoms *headache* and *abdominal pain*. For these analyses data from both cohorts, 2000 and 2008, were used. We handled the potential cohort difference by adjusting for year. In the association analyses, between pain symptoms and final school grades, *parental origin* and *parental education* were assumed to be confounders, Fig 1.

Among both girls and boys, reporting at least one of the two symptoms, compared to none, were associated with decreased school grades, Table 3.

**Table 3:** The association between having *pain symptoms* (headache, abdominal pain or both) and the level of *school grades*.

	Girls			Boys		
	Estimated school grade (95% CI)		Difference (95% CI)	Estimated school grade (95% CI)		Difference (95% CI)
	Symptom free	Any pain symptom	symptom(1)–symptom(0)	Symptom free	Any pain symptom	symptom(1)–symptom(0)
**10% percentile**						
Model 1^a^	176 (175.0 ; 176.2)	149 (148.0 ; 149.4)	-27 (-27.8 ; -26.0)	155 (154.1 ; 155.1)	128 (127.1 ; 129.1)	-27 (-27.6 ; -25.5)
Model 2^b^	172 (171.7 ; 173.2)	149 (148.0 ; 149.5)	-24 (-25.1 ; -22.7)	156 (155.3 ; 156.5)	134 (132.7 ; 135.1)	-22 (-23.3 ; -20.6)
**50% percentile**						
Model 1^a^	241 (240.1 ; 241.0)	221 (220.6 ; 221.9)	-19 (-20.1 ; -18.5)	212 (211.2 ; 212.2)	196 (195.0 ; 197.0)	-16 (-16.8 ; -14.6)
Model 2^b^	241 (240.7 ; 241.8)	221 (220.7 ; 222.2)	-20 (-20.8 ; -18.9)	212 (211.1 ; 212.1)	198 (196.6 ; 198.6)	-14 (-15.1 ; -12.9)
**90% percentile**						
Model 1^a^	298 (297.2 ; 297.9)	286 (285.8 ; 286.9)	-11 (-11.8 ; -10.6)	276 (275.3 ; 276.1)	259 (258.3 ; 260.3)	-16 (-17.5 ; -15.3)
Model 2^b^	297 (296.9 ; 297.6)	285 (284.0 ; 285.2)	-13 (-13.4 ; -11.8)	274 (273.2 ; 274.1)	260 (259.3 ; 261.5)	-13 (-14.4 ; -12.1)

Data were analyzed using quantile regression using data from both cohorts, 2000 and 2008, in 10% percentile, 50% percentile and in 90% percentile of school grades.

^a^ Model 1 is adjusted for year (2000 and 2008), parental education and foreign origin. N_Girls_ = 9889, N_Boys_ = 9685.

^b^ Model 2 is adjusted as model 1, and in addition for sedentary behavior. N_Girls_ = 9781, N_Boys_ = 9595.

Even if present in all percentiles, the decrease was most severe in the lowest percentile (10^th^), where it was equivalent to having failed in two to three out of 16 subjects (10^th^ percentile), Table 3. The magnitude of the effect slightly decreased when adjusting for sedentary behavior for the 10^th^ percentile.

Both single symptoms and combinations of symptoms predicted lower school grades, Table 4.

**Table 4:** The association between the variable *pain categories* and *school grades*.

	Difference in school grades between those with symptoms and the symptom free Estimated difference (95% CI)					
	Girls			Boys		
	Only headache	Only abdominal pain	Headache and abdominal pain	Only headache	Only abdominal pain	Headache and abdominal pain
**10% percentile**						
Model 1^a^	-12 (-12.9 ; -10.7)	-27 (-28.2 ; -25.0)	-58 (-59.6 ; -57.1)	-17 (-18.8 ; -16.0)	-5 (-6.9 ; -3.0)	-94 (-95.6 ; -92.4)
Model 2^b^	-8 (-9.6 ; -6.7)	-22 (-24.6 ; -20.3)	-50 (-52.2 ; -48.7)	-20 (-21.4 ; -18.1)	-7 (-9.5 ; -4.8)	-88 (-90.3 ; -86.5)
**50% percentile**						
Model 1^a^	-13 (14.2 ; -12.2)	-18 (-19.5 ; -16.5)	-34 (-35.7 ; -33.0)	-16 (-17.6 ; -14.8)	-5 (-7.0 ; -3.2)	-24 (-26.4 ; -21.7)
Model 2^b^	-13 (-13.7 ; -11.5)	-16 (-17.4 ; -14.0)	-32 (-34.1 ; -31.0)	-15 (-16.2 ; -13.5)	-7 (-8.6 ; -4.8)	-25 (27.3 ; -22.5)
**90% percentile**						
Model 1^a^	-9 (-9.4 ; -7.8)	-10 (-11.5 ; -9.0)	-19 (-20.3 ; -17.9)	-17 (-18.7 ; -15.8))	-7 (-8.5 ; -4.9)	-25 (-27.6 ; 22.3)
Model 2	-10 (-11.2 ; -9.4)	-11 (-12.3 ; -9.5)	-21 (-22.8 ; -20.0)	-16 (-17.3 ; -14.2)	-5 (-6.9 ; -3.0)	-26 (-29.0 ; -23.2)

The association is investigated with a quantile regression, using data from both cohorts, 2000 and 2008, in 10% percentile, 50% percentile and in 90% percentile of school grades.

^a^ Model 1 is adjusted for year (2000 and 2008), parental education and foreign origin. N_Girls_ = 9889, N_Boys_ = 9685.

^b^ Model 2 is adjusted as model 1 and in addition also for sedentary behavior. N_Girls_ = 9781, N_Boys_ = 9595.

Among both girls and boys, the effect of symptoms on grades seemed to be largest in the lowest percentile of school grades (10^th^ percentile). Hence, those with already low grades seemed to be more vulnerable. As expected school grades seemed most affected in the group with both headache and abdominal pain. Interestingly, school grades for girls were clearly more affected, than for boys, among those with abdominal pain. Also, abdominal pain in combination with headache showed the largest impact on school grades. Noteworthy in the 10^th^ percentile, boys with both headache and abdominal pain school grades had 88 to 94 lower grades than for boys with none of these symptoms. Hence, boys with already low school grades (10^th^ percentile) seemed to be a specifically vulnerable group.

The associations did not change, or only to a small extent, when adjusting for the different possible confounders (Tables 3 and 4).

### Effect modification

**Fig 2:**
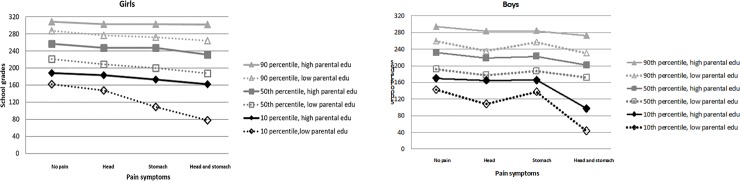
Graphical presentation of the interaction between *pain symptoms* and *parental education*. Models were adjusted for *year* and *parental origin*. *Parental education* is an effect modifier (interaction is present). The effect of pain symptoms, on school grades, is different when parents has high compared with low education. This is for example seen in the 10^th^ percentile, where the two lines for high respectively low parental education are not parallel. N_girls_ = 9889, N_boys_ = 959.

Effect modification by parental education was present among both boys and girls, with only the exception of the association between *only abdominal pain* and school grades for boys, Table 5 and Fig 2.

**Table 5:** The modification of *parental education* on the association between the variable *pain categories* and *school grades*.

	Difference in school grades between those with symptoms and the symptom free Estimated difference (95% CI)					
	Girls			Boys		
	Only headache	Only abdominal pain	headache and abdominal pain	Only headache	Only abdominal pain	headache and abdominal pain
**10% percentile**						
Low parental education	-15 (-15.5 ; -14.5)	-53 (-54.2 ; 52.6)	-85 (-85.4 ; 84.2)	-35 (-36.1 ; -33.7)	-5 (-7.2 ; -3.7)	-100 (-101.9 ; 97.9)
High parental education	-5 (-5.6 ; -4.7)	-15 (-15.9 ; -14.6)	-26 (-26.6 ; -25.3)	-6 (—6.7 ; -4.5)	-5 (-6.0 ; -3.0)	-73 (-74.6 ; -70.8)
**50% percentile**						
Low parental education	-12 (-12.6 ; -11.9)	-21 (-21.2 ; -20.2)	-33 (-33.8 ; -32.9)	-15 (-15.3 ; -14.8)	-5 (-5.1 ; -4.4)	-20 (-20.9 ; -20.0)
High parental education	-9.6 (-9.9 ; -9.4)	-10 (-10.4 ; 9.7)	-25.6 (-25.9 ; -25.3)	-14.5 (-14.7 ; -14.3)	-9 (-9.5 ; -8.9)	-30 (-30.7 ; -29.9)
**90% percentile**						
Low parental education	-10.6 (-10.8 ; -10.4)	-15 (-15.6 ; 15.1)	-23 (-23.1 ; -22.6)	-24 (-23.8 ; -23.3)	-2 (-2.5 ; 1.5)	-29 (-29.4 ; -28.3)
High parental education	-5.3 (-5.4 ; -5.2)	-5.2 (-5.3 ; -5.1)	-10.5 (-10.6 ; -10.4)	-10.6 (-10.7 ; 10.6)	-9.8 (-9.9 ; 9.7)	-20.3 (-20.4 ; 20.2)

The association is investigated with a quantile regression, using data from both cohorts, 2000 and 2008, in 10% percentile, 50% percentile and in 90% percentile of school grades. N_Girls_ = 9889, N_Boys_ = 9685. The models are adjusted for year (2000 and 2008) and foreign origin.

## Discussion

In our two study populations, of schoolchildren aged 15, the prevalence of abdominal pain increased between 2000 and 2008, most markedly in girls. The prevalence of headache also increased, but only in girls. The prevalence of pain in upper body remained similar in girls, while decreasing in boys over the 8-year period. Overall, the trends of prevalence of pain symptoms depended on type of symptom and gender. In the current study, prevalence of headache and abdominal pain were similar to previous reports [4, 34]. The prevalence for pain in upper body, ranging from 50% to 64%, were higher than prevalence of musculoskeletal pain observed in other studies [1, 3]. One possible reason for the high prevalence in the present study is that we report lifetime prevalence, while other studies usually report prevalence over shorter periods. Hence, lifetime prevalence will be higher with increasing age. Another explanation could be that we assess pain of all durations and several locations in one prevalence.

School grades were significantly lower among those with headache or abdominal pain, compared with those without any of these symptoms. Associations were strongest when both symptoms were present, and strongest in the group of students with low school grades (10^th^ percentile). Chronic pain affects cognitive function such as attention and memory in adults [35, 36]. However, the nature or consequences of cognitive disruption in schoolchildren with recurrent headache, abdominal and musculoskeletal pain is less known. A review from 2010 suggested that chronic pain affects cognitive function in children but the effects on children's function and developmental trajectories is still not yet clear [37]. Most of previous studies show that pain is associated with increased school absenteeism among children [38]. Being absent from school has the potential to affect cognitive development, but also school grades. One recent study showed that children with chronic pain in at least one body site were a year behind their peers in both reading and numeracy [20]. Our results also indicate possible serious negative impact of pain symptoms on academic achievement, in terms of final school grades. However, studies exploring direct effects of pain on children’s cognitive performance are still limited.

Among students with low school grades (10% percentile) the effect of pain symptoms were modified by parental education, i.e. low parental education was associated with severely decreased school grades in those having both headache and abdominal pain. To our knowledge, this is the first study exploring the modifying effect of low parental education on the associations between pain symptoms and school grades. This finding may be important to highlight, as it would imply a vulnerability of the students in two aspects, both due to pain symptoms and due to having parents with low educational level. The latter, it could be argued, should not occur in a community based presumed justice school system as in Sweden. Our finding of a general association between parental education and school performance is in good agreement with previous studies. A review confirmed that low socioeconomic status, such as low parental education or parental income, negatively affected the children’s academic achievements [39]. One possible explanation for these associations could be due to lower levels of engagement in child’s education among parents with lower educational level [40].

Sedentary behavior could be either a confounder or mediator for the associations between pain symptoms and school grades: physically activity may prevent symptoms of pain and pain may decrease physical activity. In a similar way, sleeping behavior can also be both a confounder and a mediator in the present study. Pain and sleep habits are strongly associated, working in both directions. In our analyses, we have considered sedentary behavior and reported sleep problems as potential confounders (by adjusting for them in a multivariate model) or as effect modifiers (in an interaction analysis). However, they could also be mediators, in which case adjusting for them would underestimate the total effect of pain symptoms on school grades. Altogether sedentary behavior and sleep problems appeared not to be confounders, as they did not substantially affect the estimated prevalence or estimated associations.

Perceived stress is a known factor associated both with neck pain, headache and abdominal pain [4]. It has been suggested that the amygdala has an essential role by coupling primary inducers of stress with somatic states [41]. Unfortunately, the present questionnaire lacked questions about stress, usable for confounding adjustment. We could therefore not adjust for stress, neither as generally perceived stress nor for a stressful home situation, e.g. economic problems, relational problems between parents, sick-listed or unemployed parents.

The current study took advantages of the two independent, large cross-sectional studies targeting all 15-year-old adolescents in all municipalities in West Sweden, the third largest county in Sweden. For both surveys, we used identical study designs, distributing the questionnaire in the same season (autumn) to same age group and using identical questions regarding pain.

Another strength of the present study is that we studied trends of both single and multiple pain symptoms among adolescents over an 8-year period. An additional strength is also that we applied a powerful statistical method, quantile regression both for handling of bounded data, and specifically for opening the possibility of studying specific subgroups as the lower 10th percentile (school grade distribution) of students.

Potential methodological limitations of our study are that the response rate was around 50% in both surveys and external missing had a higher proportion of respondents with foreign background in data from 2000, but particularly in data from 2008. We cannot exclude the possibility of selection bias concerning the higher level of parents’ education and lower participation among those of foreign descent in 2008 compared to 2000. Another limitation could be that the pain variable used in this study included not only neck but also hands and arms. In the present study, it could have been more interesting if we had only asked about neck pain, as that is the location most strongly correlated to perceived stress and psychosomatic symptoms [42]. On the other hand, the prevalence of pain in hand and arms is lower, in many populations, then in the neck [43]. Another limitation with the pain variable is that it measures musculoskeletal pain perceived ever. This might overestimate the prevalence of present symptoms. It should however be noted that the observed increase between 2000 and 2008 should not be affected by this possible misclassification as the same question is used at both time points. Hence, there is an increase in reported musculoskeletal pain in the upper body between the years 2000 and 2008, even if it is not clear whether this increase happens close to the time for the questionnaire or earlier in the children’s life. Because pain in upper body was a lifetime prevalence in this study, it was not assumed as a relevant measure for a risk factor for final school grades.

There are some methodological issues in studying the effects of pain symptoms on an adolescent’s educational outcomes. This is at least partly due to the many factors that could affect school grades such as for example general cognitive function, school absenteeism related to other than pain symptoms, mental disorders, behavioral and physiological measures. Future studies need to address these issues to obtain a better understanding of how those factors influence the associations between chronic pain and cognitive function together with academic achievement in children and adolescents.

In adults, brain imaging techniques have been used in a couple of studies to examine cognitive function in patients with chronic pain revealing for example decreased gray matter densities [15]. Future studies need to explore this issue also in younger populations since this could be seriously harmful to a developing brain.

Since multiple pain symptoms during adolescence also affects adult life [44], it is important to identify these groups of students with persistent or increasing pain problems early, for potential targeting of preventive and therapeutic interventions. We believe that it may be of great importance that the school health care early identifies students with headache, abdominal pain and combinations of symptoms. Our data suggests that actions may be of major importance, especially for students with low school grades, as co-morbidities and additional risk factors add up to worsen the problem for this vulnerable group. This is also an equality issue as children with parents with low education had a tendency of a stronger negative impact of the pain symptoms on school grades, than children with highly educated parents.
